# Supplementary material for: Class IIa HDACs regulate learning and memory through dynamic experience-dependent repression of transcription
Source: Nat Commun. 2019 Aug 2;10:3469. doi: 10.1038/s41467-019-11409-0 (PMC6677776; doi:10.1038/s41467-019-11409-0)
Supplement: Supplementary file 1 — Supplementary Information [file 41467_2019_11409_MOESM1_ESM.pdf]

Supplementary information for Zhu el al.,
"Class IIa HDACs regulate learning and memory through dynamic experience-dependent repression of transcription"

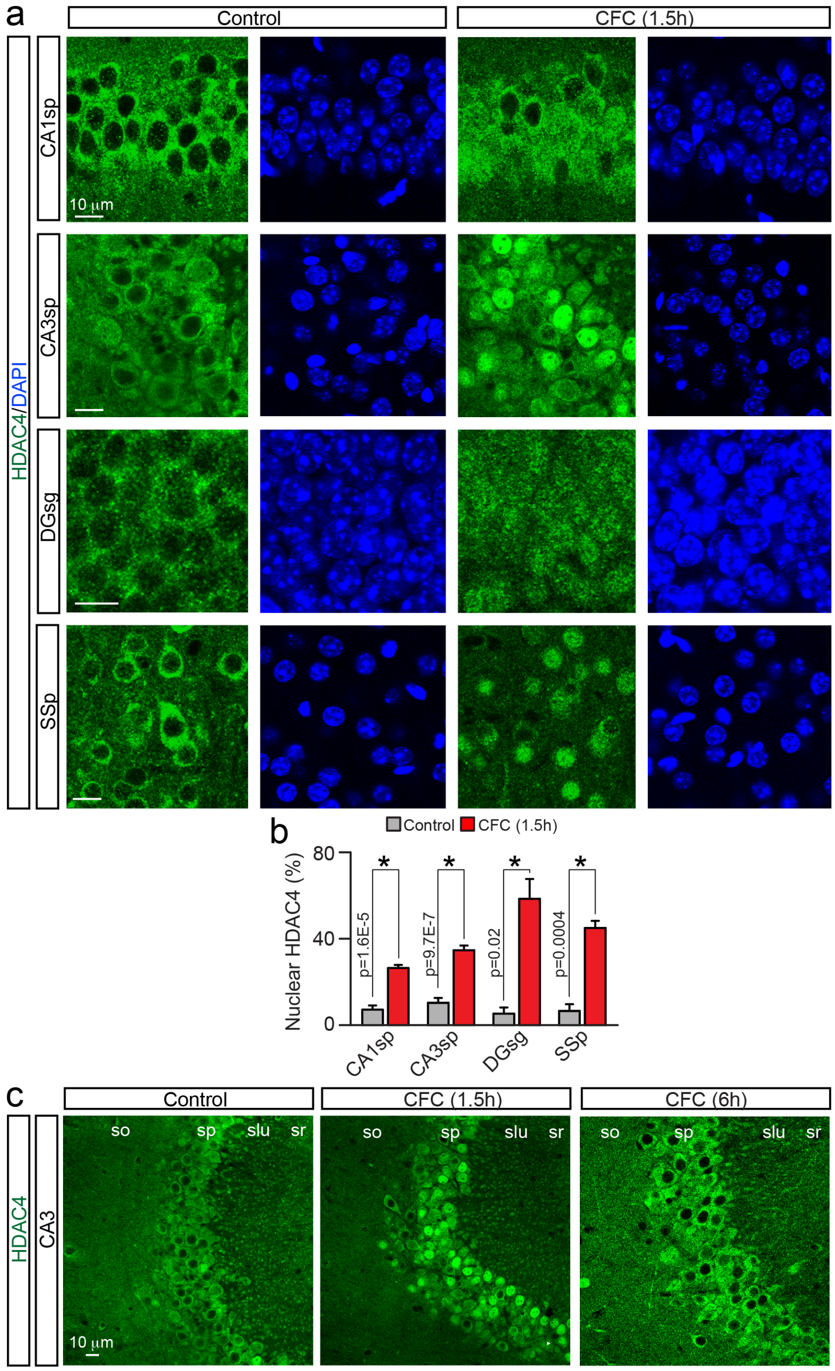

Supplementary Figure 1 | Subcellular localization of native HDAC4 in brains of normal and fear-conditioned mice. Brain sections were labeled with the antibody against HDAC4 and a nuclear marker, DAPI. **a,** Typical high magnification confocal images of CA1sp, CA3sp, DGsg and SSp in samples from normal and foot-shocked animals (1.5 hours post-CFC). **b,** Averaged percentages of cells with nuclear HDAC4 in indicated areas. Total cell numbers were quantified with DAPI. *n* = 3-4 mice/group. **c,** Images of the CA3 demonstrate than nuclear accumulation of HDAC4 during associative learning is reversible.

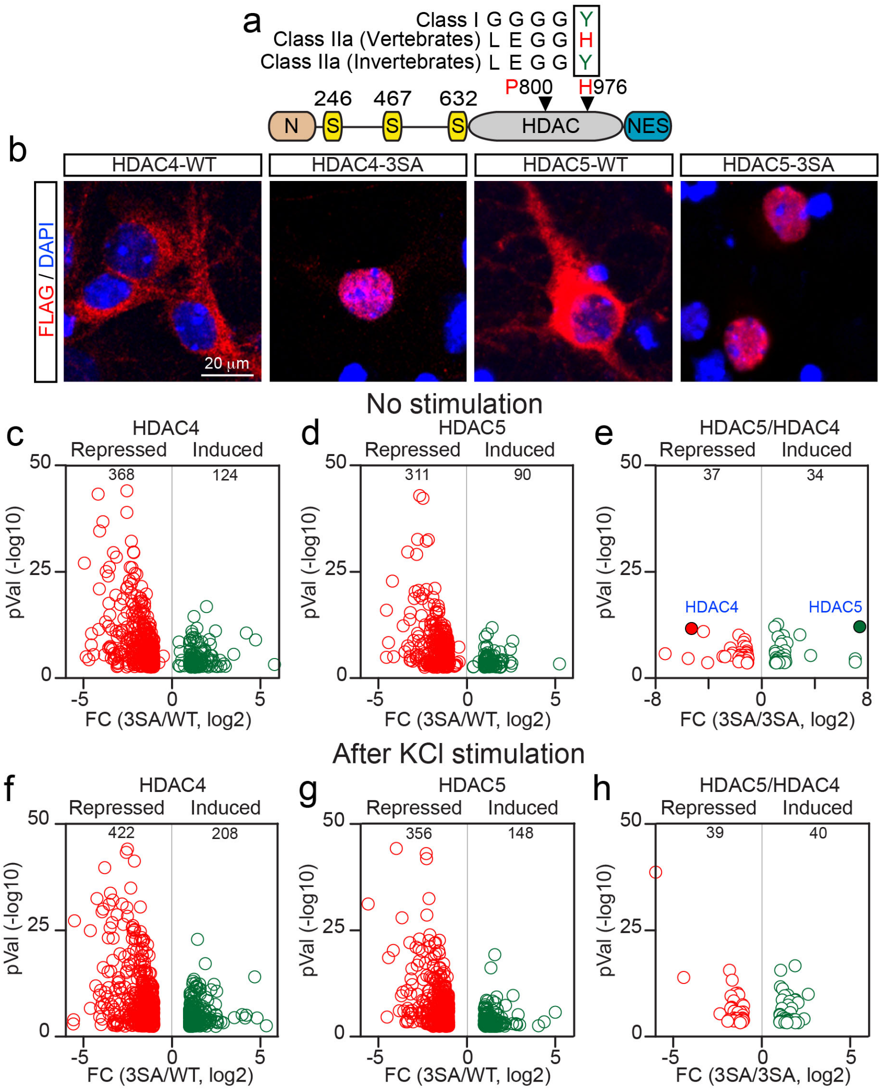

Supplementary Figure 2 | a, Domain structure of HDAC4 (based on human sequence). The diagram depicts the N-terminal region, Serine residues 246, 467 and 632 whose phosphorylation promotes nuclear export, histone deacetylase domain (HDAC), nuclear export signal (NES), a frame shift mutation associated with mental retardation in humans (P800), and a mutation in the active site that diminishes catalytic activity on ε-N-acetyl lysine substrates (H976). A sequence alignment of these coding regions of conventional class I HDACs, and invertebrate and vertebrate class IIa HDACs is also shown (see also Lahm et al., 2007, Williams et al., 2010 and Sando et al., 2012). **b-h,** Analysis of nuclear repressor activity of HDAC4 and HDAC5 *in vitro*. FLAG-tagged wildtype class IIa HDACs or their constitutively nuclear phosphorylation-deficient mutants (3SA) were broadly expressed in cortical neurons in primary cultures from lentiviruses under the control of the Synapsin promoter. Cultures were infected at 2 days *in vitro* (DIV2) and analyzed at DIV7. **b,** Localization of recombinant proteins. Confocal images of neurons that were labeled with DAPI and the antibody against FLAG are shown. **c-h,** Gene expression levels were examined by deep sequencing (RNA-seq) in unstimulated (**c-e**) and KCl-depolarized neurons (**f-h**). Volcano plots of all differentially expressed transcripts from 2 independent RNA-seq experiments are shown (total numbers are indicated in each panel). In panels **c, d, f** and **g**, fold changes (FC) are represented as log2 of 3SA/WT ratio for each HDAC isoform. Negative and positive values reflect repression and upregulation by 3SA mutants, respectively. Panels **e** and **h** demonstrate that only few genes are differentially expressed in neurons carrying HDAC4-3SA and HDAC5-3SA.

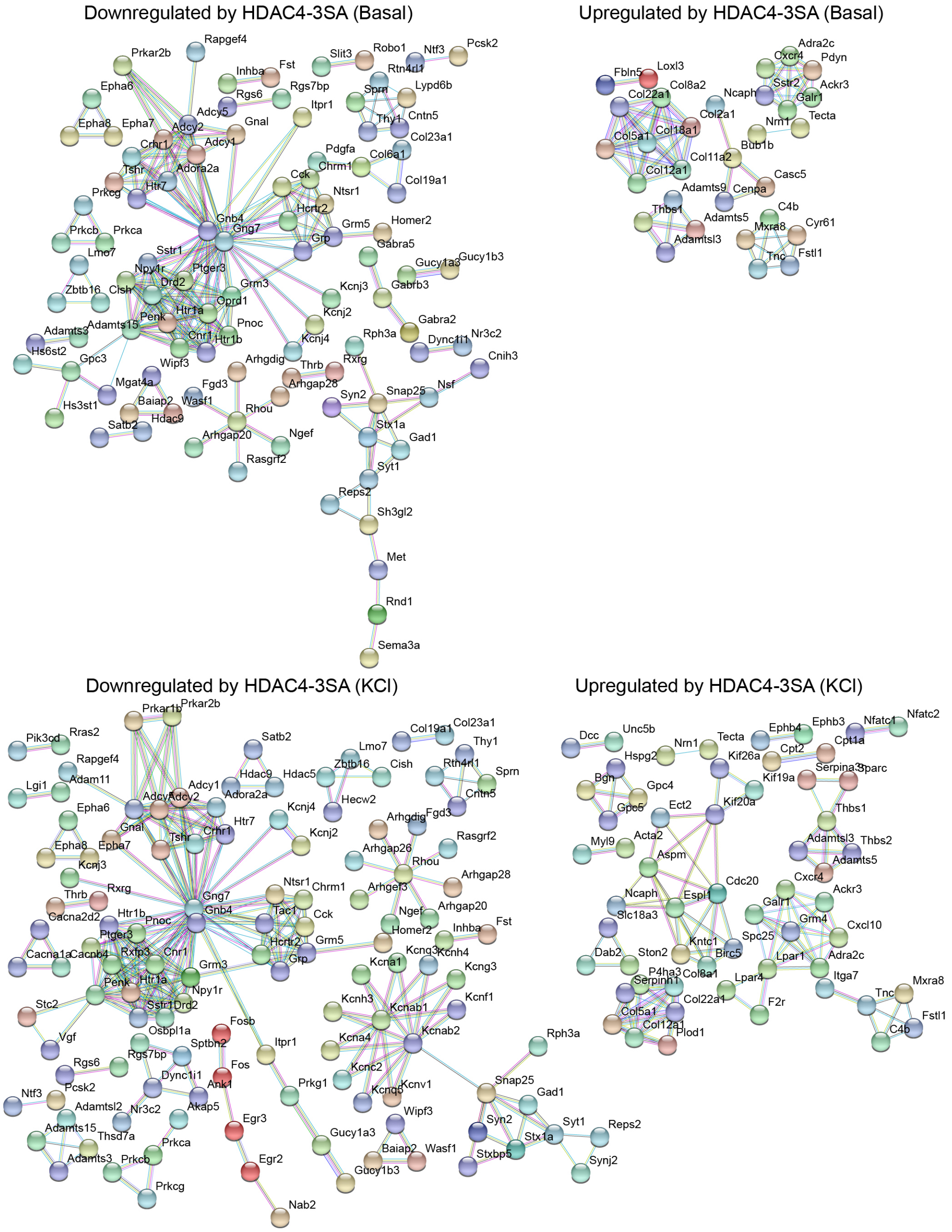

Supplementary Figure 3 | STRING pathway analysis of genes that are differentially expressed in cultured neurons carrying the constitutively nuclear HDAC4-3SA mutant. Networks were generated at https://string-db.org using the highest confidence interaction score of 0.9. Disconnected nodes are not displayed. Full lists of genes, including genes that are mis-regulated in the presence of HDAC5-3SA can be found in accompanying Supplementary Table 1.

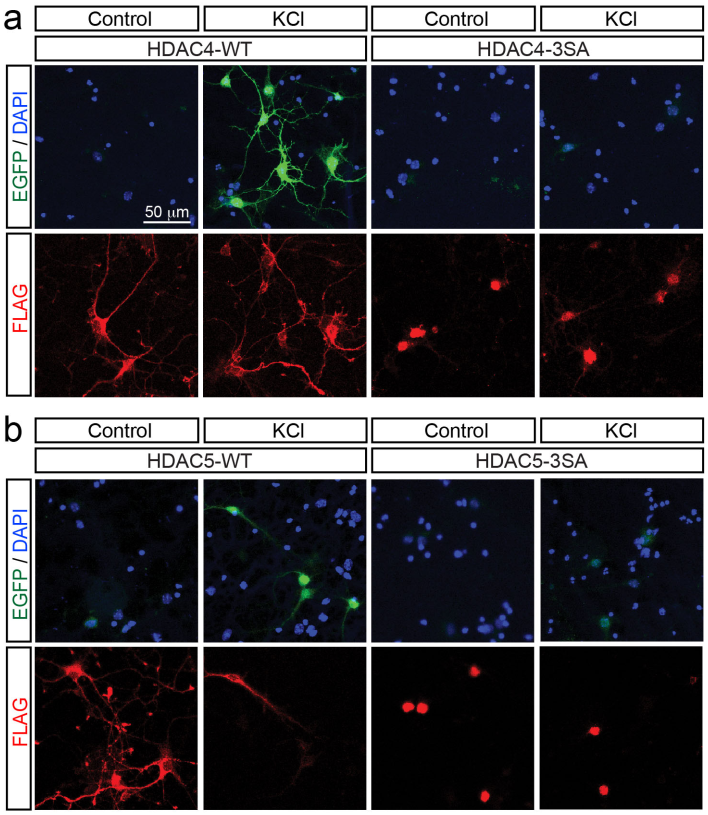

Supplementary Figure 4 | Nuclear class IIa HDACs repress an activity-dependent reporter, E-SARE:EGFP. a, Confocal images of unstimulated and KCl-depolarized neurons that co-expressed E-SARE:EGFP with either cytoplasmic wildtype of nuclear gain-of-function 3SA HDAC4 construct. **b,** Confocal images of unstimulated and KCl-depolarized neurons that co-expressed E-SARE:EGFP with either wildtype of nuclear gain-of-function form of HDAC5. In both panels, cultures were labeled with an antibody against FLAG and DAPI.

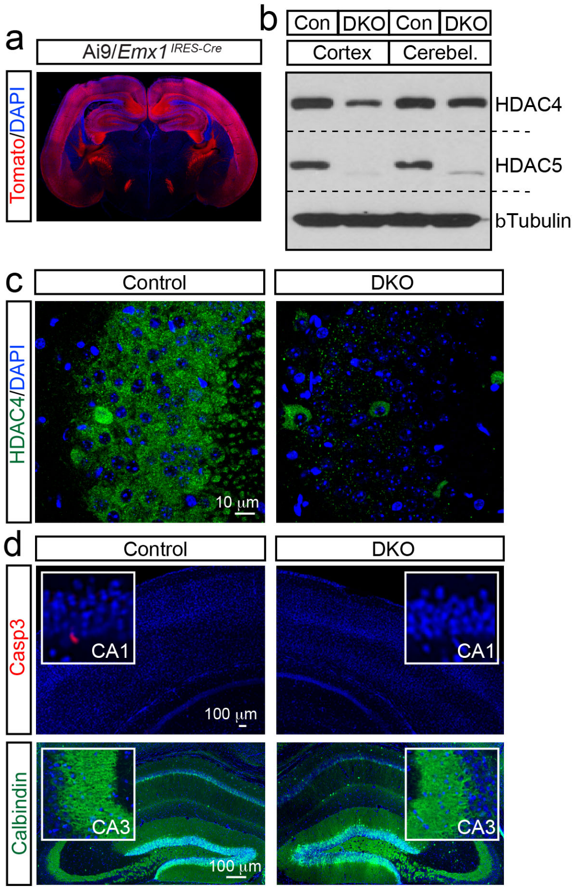

Supplementary Figure 5 | a, Expression of tdTomato Cre reporter in *Ai9/Emx1*$^{IRES\text{-}Cre}$ mice. For detailed analysis, see Sando et al., Neuron, 2017. **b,** Total protein homogenates from cortices and cerebella of adult wildtype (Control) and *Hdac4*$^{flox/flox}$/*Hdac5*$^{-/-}$/*Emx1*$^{IRES\text{-}Cre}$ (DKO) mice were probed by immunoblotting with antibodies against HDAC4, HDAC5 and βTubulin (as a loading control). Note that remaining expression of HDAC4 in DKO forebrain reflects lack of recombination in inhibitory GABAergic interneurons. **c, d,** Brain sections from p60 control and DKO mice were labeled with DAPI and antibodies against HDAC4 (**c**) or cleaved Caspase 3 and Calbindin (**d**). Representative confocal images are shown.

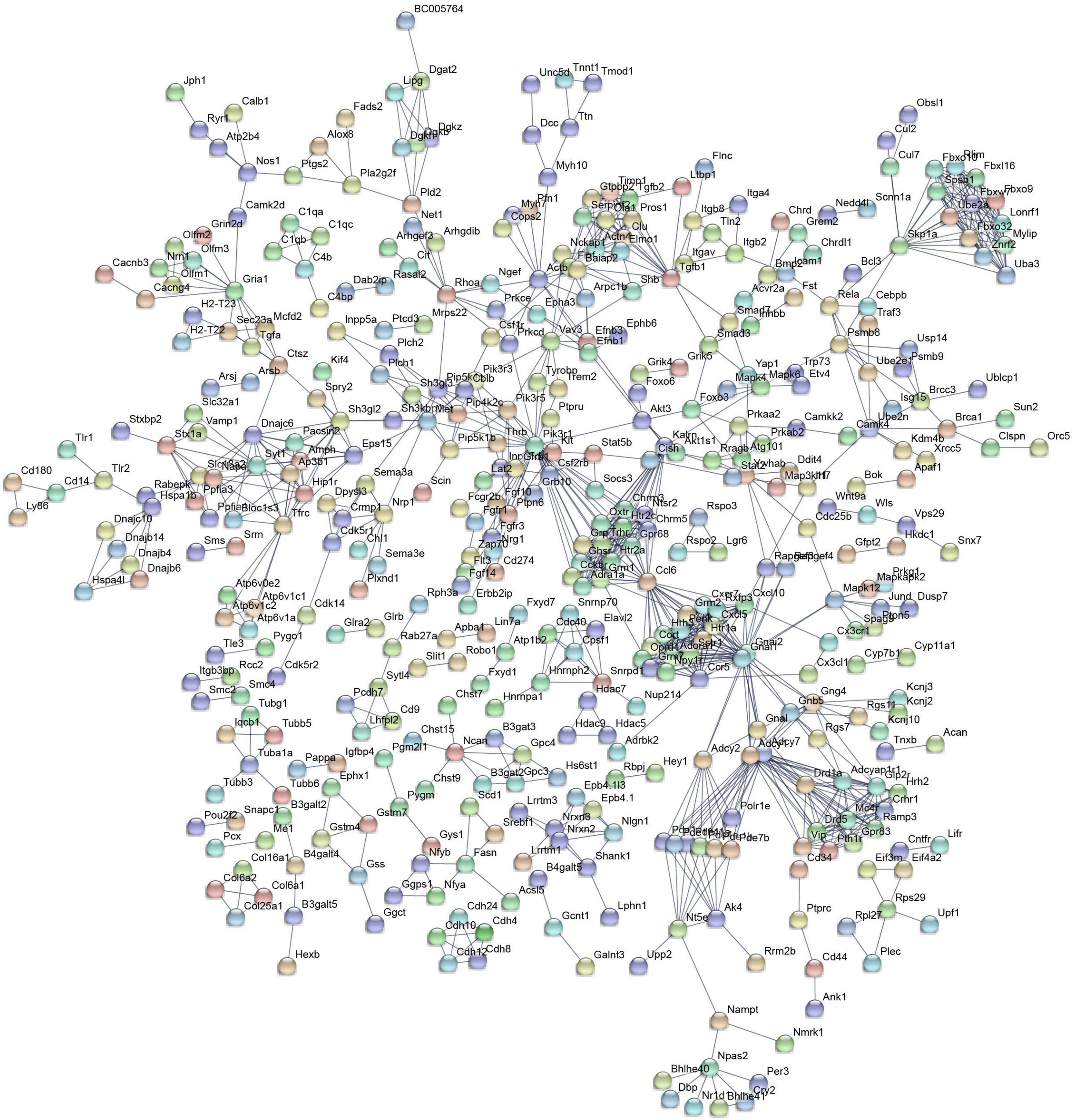

Supplementary Figure 6 | STRING pathway analysis of differentially expressed genes in the hippocampus of DKO mice. The network was generated at https://string-db.org using the highest confidence interaction score of 0.9. Disconnected nodes are not displayed.

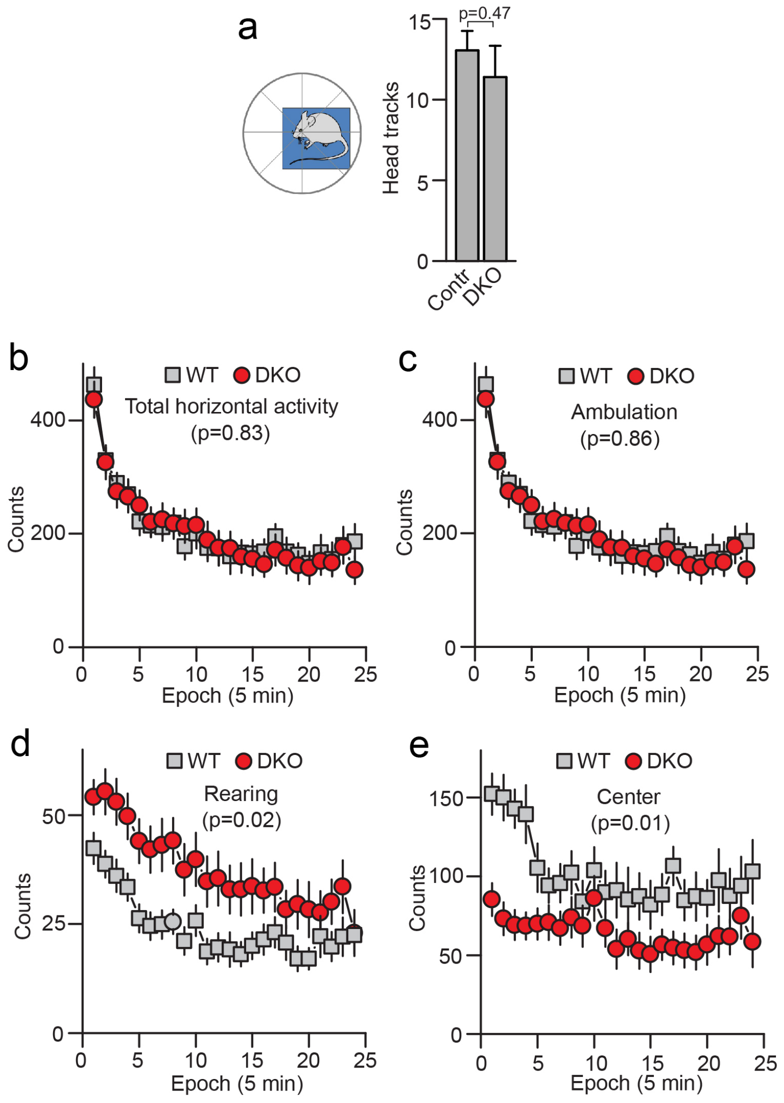

Supplementary Figure 7 | a, Analysis of vision of p60 control and DKO mice. Schematic representation of the optomotor setup and averaged head tracks are shown. Control: $n = 19$; DKO: $n = 18$. **b-e,** Detailed analysis of behavior in the open field. Quantifications of total horizontal activity (**b**), ambulation (**c**), rearing (**d**) and center activity (**e**) are shown. Control: $n = 24$; DKO: $n = 24$. All graphs are plotted as Mean ± S.E.M. p values were determined by t-test.

a

MISLIAALAVDYVIGMENAMPWNLPADLAWFKRNTLNKPVIMGRYTWESIGRPLPGRKNIILSSQPSTDDRVTWV
KSVDEAIAACGDVPEIMVIGGGRVIEQFLPKAQKLYLTHIDAEVEGDTHFPDYEPDDWESVFSEFHDADAQNSHSY
CFEILERRGAPMSSQSHPDGLSGRDQPVELLNPARVNHMPSTVDVATALPLQVAPSAVPMDLRLDHQFSLPVAEP
ALREQQLQQELLALKQKQQIQRQILIAEFQRQHEQLSRQHEAQLHEHIKQQQEMLAMKHQQELLEHQRKLERHR
QEQELEKQHREQKLQQLKNKEKGKESAVASTEVKMKLQEFVLNKKKALAHRNLNHCISSDPRYWYGKTQHSSLDQ
SSPPQSGVSTSYNHPVLGMYDAKDDFPLRKTAAEPNLKLRSRLKQKVAERRSSPLLRRKDGPVVTALKKRPLDVTDS
ACSSAPGSGPSSPNNSSGSVSAENGIAPAVPSIPAETSLAHRLVAREGSAAPLPLYTSPSLPNITLGLPATGPSAGTAG
QQDAERLTLPALQQRLSLFPGTHLTPYLSTSPLERDGGAAHSPLLQHMVLLEQPPAQAPLVTGLGALPLHAQSLVG
ADRVSPSIHKLRQHRPLGRTQAAPLPQNAQALQHLVIQQQHQQFLEKHKQQFQQQQLQMNKIIPKPSEPARQPE
SHPEETEEELREHQALLDEPYLDRLPGQKEAHAQAGVQVKQEPIESDEEEAEPPREVEPGQRQPSEQELLFRQQAL
LLEQQRIHQLRNYQASMEAAGIPVSFGGHRPLSRAQASPASATFPVSVQEPPTKPRFTTGLVYDTLMLKHQCTCGS
SSSHPEHAGRIQSIWSRLQETGLRGKCECIRGRKATLEELQTVHSEAHTLLYGTNPLNRQKLDSKKLLGSLASVFVRL
PCGGVGVDSDTIWNEVHSAGAARLAVGCVVELVFKVATGELKNGFAVVRPPGHHAEESTPMGFCYFNSVAVAAK
LLQQRLSVSKILIVDWDVHHGNGTQQAFYSDPSVLYMSLHRYDDGNFFPGSGAPDEVGTGPGVGFNVNMAFTG
GLDPPMGDAEYLAAFRTVVMPIASEFAPDVVLVSSGFDAVEGHPTPLGGYNLSARCFGYLTKQLMGLAGGRIVLAL
EGGHDLTAICDASEACVSALLGNELDPLPEKVLQQRPNANAVRSMEKVMEIHSKYWRCLQRTTSTAGRSLIEAQTC
ENEEAETVTAMASLSVGVKPAEKRPDEEPMEEEPPLEFGGDYKDDDDK*

Blue: ecDHFR (DD)
Black: HDAC4 (S/A mutations are shown in red)
Yellow: FLAG tag

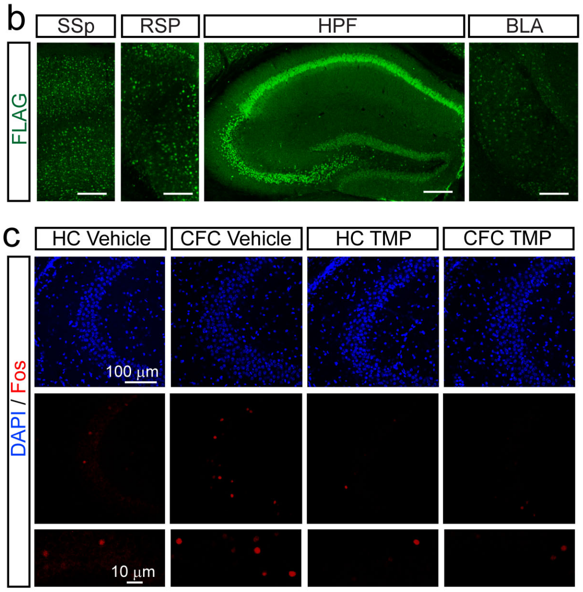

Supplementary Figure 8 | Characterization of *R26$^{floxStop\text{-}DD\text{-}nHDAC4}$*/*Camk2$\alpha^{Cre}$* mice. a, Primary aminoacid sequence of DD-nHDAC4 fusion protein. **b,** Immunofluorescent imaging analysis of expression of DD-nHDAC4-F in different brain regions. SSp: Primary somato-sensory cortex; RSP: Retrosplenial cortex; HPF: Hippocampus; BLA: Basolateral amygdala. **c,** Acutely stabilized DD-nHDAC4 represses ERGs. *R26$^{floxStop\text{-}DD\text{-}nHDAC4}$*/*Camk2$\alpha^{Cre}$* mice were given single doses of vehicle or TMP. Injected animals were either maintained in home cages (HC) or subjected to associative learning via CFC (3 hours post-injection). 1.5 hours later, brains were isolated, sectioned, and imaged after labeling with DAPI and an antibody against Fos. Typical images of the CA3 are shown.
