# Supplementary material for: Class IIa HDACs regulate learning and memory through dynamic experience-dependent repression of transcription
Source: Nat Commun. 2019 Aug 2;10:3469. doi: 10.1038/s41467-019-11409-0 (PMC6677776; doi:10.1038/s41467-019-11409-0)
Supplement: Supplementary file 4 — Description of Additional Supplementary Files [file 41467_2019_11409_MOESM4_ESM.docx]

**Description of Additional Supplementary Files**

File Name: Supplementary Data File 1
Description: RNA-seq analysis of gene expression in cultured neurons

File Name: Supplementary Data File 2
Description: scRNA-seq analysis of hippocampi of control and DKO mice

File Name: Supplementary Data File 3
Description: RNA-seq analysis of gene expression in hippocampi of control and DKO mice.
