# Supplementary material for: Class IIa HDACs regulate learning and memory through dynamic experience-dependent repression of transcription
Source: Nat Commun. 2019 Aug 2;10:3469. doi: 10.1038/s41467-019-11409-0 (PMC6677776; doi:10.1038/s41467-019-11409-0)
Supplement: Supplementary file 2 — Peer Review [file 41467_2019_11409_MOESM2_ESM.pdf]

Reviewers' comments:

Reviewer #1 (Remarks to the Author):

In their paper, Class IIa HDACs regulate learning and memory through dynamic experience-dependent repression of transcription, Zhu et al. address the repressive role that the HDACs 4 and 5 play to maintain function during learning. They do this by first characterizing normal HDAC4 in the mouse hippocampus during contextual fear conditioning (CFC) and then by characterizing two HDAC4/5 mutants, one that constitutively expresses both HDAC4 and HDAC5 (3SA) in cell nuclei and another double knockdown (DKO).

First, they characterize HDAC4 localization in the mouse brain after contextual fear conditioning (CFC) (Fig 1), showing that not only does HDAC4 translocate from the cytoplasm to nuclei between 1.5 and 6 hours after CFC, but also that this translocation does not occur in cells expressing Fos. They further characterize HDAC4 and 5 effects by creating an HDAC4/5 (3SA) mutant that is constitutively localized to the nucleus and showing that these mutants have reduced expression of early response genes (ERGs) in response to cell activation (using KCl). Zhu et al then look at this in vivo by creating mice with a knockdown of HDAC4 and HDAC5 (DKO) that show a general increase in anxiety and a decrease in memory (single knockouts yield no such effects).

In the second half of this manuscript, Zhu et al. try to further classify DKO mice. First, they find that, while there is no neuronal structure loss, there is an increase in post-synaptic spines in the glutamatergic neurons. They also show, using electrophysiological experiments that there is an increase in frequency and amplitude of EPSCs in the DKO mice, suggestion higher numbers of excitatory inputs, thus increasing LTP. Finally, they show in vivo that the presence of the destabilized HDAC4-3SA in the nucleus silences Egr1 and Fos expression in the CA3 after fear conditioning and that when this is HDAC4-3SA is expressed before fear conditioning animals show a reduction in freezing, whereas when this is done before recall, there is no effect, claiming that HDAC4 transcriptional repression plays a role in memory acquisition but not memory recall.

Overall, this is an intriguing study that adds to the growing literature on the role of HDACs in memory and cognition. The experiments appear to be sound, and the paper is a good fit for Nature Communications, provided that the following major and minor revisions are addressed.

Major Points
- The main argument of the paper is that the nuclear shuffling of HDAC4 and 5 accompanies memory formation. However, the authors do not show any learning effect/behavioral data in figure 1. Likewise, the expression of IEGs in Figure 2l is not correlated to the memory performance of the mice. This is critical to show.
- Furthermore, in Figure 2l, the timeline of the mRNA changes could also be attributed to non-HDAC4/5 related effects, given the timeline of the nuclear shuffling shown in figure 1a/b. The authors should clarify this point.
- Throughout the manuscript, statistics used should be clarified for each section of each figure in the figure legend. In addition, the comparisons in Figure 1B should be tested with an ANOVA instead of a t-test.
- The authors should either elaborate on or remove the data from the single cell dataset.
o In the methods, it says that they enrich for neurons before sequencing, but then they go on to compare cell types in the two samples. The cell type analysis is likely biased.
o They authors use Figure 2g to show that there are no differences in cluster size between the two datasets, but do not elaborate on what is in the clusters or include any information about the actual cell numbers or types in each cluster.
Minor Points
- Minor spelling mistakes occur throughout the manuscript and should be corrected.
- Introduction

o Tyssowski et al., Neuron, 2018 should be included in the references about waves of experience dependent transcriptional changes in the first paragraph of the introduction.
o Reference Josselyn et al, Nature Reviews Neuroscience, 2015 at the end of the second paragraph in the introduction
o The sentence "yet the functional role of HDAC4 in the central nervous system has remained enigmatic" in the introduction is not accurate, given the authors' own previous publication(s).
- Figure 1
o The referenced figures should beging in order (Supplementary Fig. 1a,b,c should come first)
o Split the channels in Figure 1a as done in Supplementary Figure 1.
o Is the difference between Control and CFC significant in Figure 1b?
o The phrase "indirectly suggesting that HDAC4 restricts the expression of Fos and possibly other ERGs in circuits essential for memory storage (Fig. 1c,d)" isn't accurate. It's possible that HDAC4 is not translocated because of ERG expression.
o Include HDAC5 panels from the supplementary data to main figure 1g.
o In Figure 1e/f, there are a few ERGs that are shown as down-regulated. I would be interested in seeing a supplementary figure that also briefly discusses if there are learning specific pathways in the up-regulated genes.
o The sentence "While ERGs were not the only effectors of HDAC4 in these artificial settings, it is unlikely that direct silencing of other constitutively expressed genes in the normal brain is physiologically relevant given the narrow window of repressor accumulation in the nucleus" is not only logically incorrect (constitutive meaning constitutive) but also not supported by any data.
- Figure 2
o It would be interesting to touch upon other pathways that are not related to learning are in the STRING network in figure 2i (and are the correlated genes up or down-regulated in the RNA-seq dataset?). This information could be used to infer other effects of HDAC4 and HDAC5.
o It is unclear why authors sometimes show data from the CA3 (Figure 2, e.g.) and other times show data from the CA1 (Figure 4, e.g.)
- Figure 3
o Figure 3i is misleading. They should show the sex differences for all groups, not just the single HDAC5 KO.
- Figures 4 and 5
o Methodological details for counting spines are missing from the manuscript.
o Clarify what is being refered to when referencing "viral tracing" in the section "HDAC4/5-deficient excitatory circuits have increased synaptic strength".
- Figure 6
o In figure 6, it is not so clear that the nHDAC4 is the HDAC4-3SA. Please indicate this in the figure.
o In figure 6, the authors show that HDAC4 stabilization affects memory acquisition but not memory recall. Are there differences in HDAC4 location between the two? It could be nice to show this in the same manner as in Figure 1A.
- Discussion
o Gräff & Tsai, 2013, Ann Rev Pharmacol and Toxicol should also be cited when referencing small molecule inhibitors of HDACs in the discussion
o Elaborate on how mechanisms of Class I HDACs differ from those of Class IIs.
o The claim that HDACs4 and 5 have an "irreversible nuclear entry" has been shown to be untrue in Figure 1a.
o The last sentence in the discussion is too strong to comfortably claim and should be toned down as it is not warranted by any data.

Reviewer #2 (Remarks to the Author):

In this manuscript the authors follow up on their 2012 Cell paper to elucidate the functions of the nuclear import of the class II HDACs (4 and 5) in the mouse brain. They generate double excitatory

neuronal knockouts of Hdac4/5 as well as a nice tool that lets them rapidly express a triple phospho-mutant form of Hdac4 that stays in the nucleus and acts as a dominant negative. Some of the results in the manuscript are rather expected, such as the nuclear-cytoplasmic translocation of Hdac4 or the evidence Hdac4 acts as a repressor, but it is still nice to see such a complete and well documented dataset showing the role of HDAC4/5 as transient repressors of IEG induction and then using that disruption to suggest the functional importance of IEGs for learning/memory behaviors.

Overall the experiments are performed to a high technical standard and the data advance understanding of the mechanisms that sculpt the pattern of activity-inducible gene expression in vivo. I have only very minor concerns.

1) The writing could be tempered in places. For example, the references to functions of Hdac4 and Hdac5, which, though perhaps not completely understood, have been published in nice papers from Lisa Monteggia, Chris Cowan, Eric Nestler, and the authors themselves among others, and should not be described as "enigmatic" or "unknown".

2)I was surprised the authors did not discuss HDAC9 since it is the third member of the Class II HDAC family. Is it expressed in the CNS and also undergoes activity-dependent shuttling (e.g. PMID 28729685) and has activity-regulated, MEF2-dependent functions. Is there some reason this factor is not also redundant with Hdac4/5?

3) Given that glutamate neurotransmission and activation of NMDARs leads to nuclear export of HDAC4, it is interesting that the authors find transient increases in nuclear HDAC4/5 following CFC in brain regions showing increased Fos expression, which is a canonical marker of "increased activity". Presumably this reflects differences in the firing of different neurons in the circuit, with those that fire most having cytoplasmic Hdac. However for readers not familiar with the past literature they may be confused and read this paper to suggest that increased neuronal activity drives the nuclear accumulation of HDAC. This could be made more clear in the text.

4) Given that there are changes in synaptic connectivity and function in the double KO mice, the authors should demonstrate that the increased IEG expression they see after CFC is really due to reduced repression of IEGs as opposed to just increased upstream neuronal activity. The easiest way to do this is actually to depolarize the double knockout neurons in culture, since this stimulus bypasses the synapses and directly activates intracellular calcium signaling pathways that normally induce nuclear export of Hdacs. As near as I can tell this experiment is non-redundant with other published experiments because no prior lab has had the double knockout mice to demonstrate the requirement for the Hdacs in the time course or overall magnitude of IEG induction.

Zhu et al., Responses to Reviewer's Comments.
Reviewer #1 (Remarks to the Author):

In their paper, Class IIa HDACs regulate learning and memory through dynamic experience-dependent repression of transcription, Zhu et al. address the repressive role that the HDACs 4 and 5 play to maintain function during learning. They do this by first characterizing normal HDAC4 in the mouse hippocampus during contextual fear conditioning (CFC) and then by characterizing two HDAC4/5 mutants, one that constitutively expresses both HDAC4 and HDAC5 (3SA) in cell nuclei and another double knockdown (DKO). First, they characterize HDAC4 localization in the mouse brain after contextual fear conditioning (CFC) (Fig 1), showing that not only does HDAC4 translocate from the cytoplasm to nuclei between 1.5 and 6 hours after CFC, but also that this translocation does not occur in cells expressing Fos. They further characterize HDAC4 and 5 effects by creating an HDAC4/5 (3SA) mutant that is constitutively localized to the nucleus and showing that these mutants have reduced expression of early response genes (ERGs) in response to cell activation (using KCl). Zhu et al then look at this in vivo by creating mice with a knockdown of HDAC4 and HDAC5 (DKO) that show a general increase in anxiety and a decrease in memory (single knockouts yield no such effects). In the second half of this manuscript, Zhu et al. try to further classify DKO mice. First, they find that, while there is no neuronal structure loss, there is an increase in post-synaptic spines in the glutamatergic neurons. They also show, using electrophysiological experiments that there is an increase in frequency and amplitude of EPSCs in the DKO mice, suggestion higher numbers of excitatory inputs, thus increasing LTP. Finally, they show in vivo that the presence of the destabilized HDAC4-3SA in the nucleus silences Egr1 and Fos expression in the CA3 after fear conditioning and that when this is HDAC4-3SA is expressed before fear conditioning animals show a reduction in freezing, whereas when this is done before recall, there is no effect, claiming that HDAC4 transcriptional repression plays a role in memory acquisition but not memory recall.

Overall, this is an intriguing study that adds to the growing literature on the role of HDACs in memory and cognition. The experiments appear to be sound, and the paper is a good fit for Nature Communications, provided that the following major and minor revisions are addressed.

We thank the reviewer for his/her positive evaluation of our work and insightful comments which helped us to substantially improve the study. In the revised paper, these comments are addressed both experimentally and textually, as outlined below.

Major Points

The main argument of the paper is that the nuclear shuffling of HDAC4 and 5 accompanies memory formation. However, the authors do not show any learning effect/behavioral data in figure 1. Likewise, the expression of IEGs in Figure 2l is not correlated to the memory performance of the mice. This is critical to show.

We agree and now include new experiments that directly correlate the localization of HDAC4 and measurements of ERG levels with memory performance in both wildtype (new Fig. 1b) and double-knockout mice (new Fig. 2j). These results confirm that transient experience-dependent nuclear import of the repressor coincides with acquisition of fear memory, as assessed by quantifications of freezing in the same context (note that imaging and behavioral studies were done at matching timepoints). It is also important to emphasize that our new measurements of short-term memory are consistent with results presented in the original version the paper: mice lacking two class IIa HDAC isoforms in glutamatergic neurons are capable of initially acquiring a fear memory but they have dramatic defects in extinction (Fig. 3h).

Furthermore, in Figure 2l, the timeline of the mRNA changes could also be attributed to non-HDAC4/5 related effects, given the timeline of the nuclear shuffling shown in figure 1a/b. The authors should clarify this point.

This point is addressed with new measurements of short-term memory performance in mutant mice, as discussed above. We also included additional clarifications in the text.

Throughout the manuscript, statistics used should be clarified for each section of each figure in the figure legend. In addition, the comparisons in Figure 1B should be tested with an ANOVA instead of a t-test.

We now provide detailed descriptions of statistics and use ANOVA in addition to t-test in Fig. 1d (original Fig. 1b). We also provide all raw data in the separate Excel spreadsheet (Source data) to comply with Nature Communications policies.

The authors should either elaborate on or remove the data from the single cell dataset. In the methods, it says that they enrich for neurons before sequencing, but then they go on to compare cell types in the two samples. The cell type analysis is likely biased. They authors use Figure 2g to show that there are no differences in cluster size between the two datasets, but do not elaborate on what is in the clusters or include any information about the actual cell numbers or types in each cluster.

We apologize for this oversight: indeed, our samples contained multiple cells types. While it is possible that our experimental procedure led to a certain bias in terms of cellular composition of the input material (we used protocols for tissue dissociation that were previously published by other labs), note that we performed side-by-side scRNA-seq analysis of normal and DKO mice. We have corrected the description of methods and added more details to elaborate the scRNA-

seq results in new Supplementary Table S2, including cell numbers and all raw data. Also note that the outcome of scRNA-seq experiments is not central to this study, but it is in agreement with IF imaging analysis of the distribution of lineage-specific markers of excitatory neurons in the cortex and hippocampus, which demonstrates that DKO mice have no detectable anatomical defects. As we discussed in the main text, we chose the standard RNA-seq for quantitative measurements of gene expression levels since the coverage of scRNA-seq is relatively shallow.

Minor Points

Minor spelling mistakes occur throughout the manuscript and should be corrected.

We tried to correct all typos throughout the text.

Tyssowski et al., Neuron, 2018 should be included in the references about waves of experience dependent transcriptional changes in the first paragraph of the introduction .... reference Josselyn et al, Nature Reviews Neuroscience, 2015 at the end of the second paragraph in the introduction

These references are now included, as recommended.

The sentence "yet the functional role of HDAC4 in the central nervous system has remained enigmatic" in the introduction is not accurate, given the authors' own previous publication(s).

We have toned down this statement, as recommended (similar point was made by Reviewer #2). We used the word "enigmatic" in the original draft since several groups argued that HDAC4 does not function as a nuclear repressor based on lack of changes in gene expression in single knockout mice. Our results demonstrate that these puzzling observations are attributed to functional redundancy and, also, the fact that repressors only undergo nuclear export in the normal brain in a transient manner.

The referenced figures should beging in order (Supplementary Fig. 1a,b,c should come first).

We now reference all figures in order, as recommended.

Split the channels in Figure 1a as done in Supplementary Figure 1.

The channels are now split (new Fig. 1c).

Is the difference between Control and CFC significant in Figure 1b?

The differences between Control and 1.5h post-CFC are statistically significant for both CA1 and CA3. The difference between Control and 6h post-CFC (when nuclear import is reversed) is only significant for CA3. We indicated this in figure legend. Note that these data are now presented in Fig.1d.

The phrase "indirectly suggesting that HDAC4 restricts the expression of Fos and possibly other ERGs in circuits essential for memory storage (Fig. 1c,d)" isn't accurate. It's possible that HDAC4 is not translocated because of ERG expression.

We have changed this sentence from "indirectly suggesting" to "raising the possibility"

Include HDAC5 panels from the supplementary data to main figure 1g.

We only included a partial set of images since this main figure is already very crowded and the space is limited. We will be happy to move other images from the supplementary figure, if our reviewer and editors insist.

In Figure 1e/f, there are a few ERGs that are shown as down-regulated. I would be interested in seeing a supplementary figure that also briefly discusses if there are learning specific pathways in the up-regulated genes.

We performed these analyses, as recommended. The STRING networks of genes that are down- or upregulated by nuclear HDAC4 under the normal conditions and after KCl stimulation are now shown separately in new Supplementary Fig. 3. Since we generated these networks using the highest confidence score of 0.9 and cut off disconnected nodes, we also provided full gene lists for both HDAC4-3SA and HDAC5-3SA in Supplementary Table 1.

The sentence "While ERGs were not the only effectors of HDAC4 in these artificial settings, it is unlikely that direct silencing of other constitutively expressed genes in the normal brain is physiologically relevant given the narrow window of repressor accumulation in the nucleus" is not only logically incorrect (constitutive meaning constitutive) but also not supported by any data.

We removed this sentence.

It would be interesting to touch upon other pathways that are not related to learning are in the STRING network in figure 2i (and are the correlated genes up or down-regulated in the RNA-seq dataset?). This information could be used to infer other effects of HDAC4 and HDAC5.

We have included a description of all major networks, as revealed by STRING, in the corresponding main figure legend. Gene names are listed in Supplementary Fig. 6. We have also provided all raw RNA-seq data in Supplementary Table 3.

It is unclear why authors sometimes show data from the CA3 (Figure 2, e.g.) and other times show data from the CA1 (Figure 4, e.g.)

We performed imaging and electrophysiology in the CA1 because synapses in the CA3 are more heterogeneous (e.g. large Mossy fiber terminals of dentate granule cells, terminals of mossy cells, recurrent connections of CA3 pyramidal neurons, and terminals of cortical axons), which makes it harder to analyze and interpret the data, especially EM tomography. Since area CA1 receives direct input from the CA3, we believe this approach is appropriate. Please also note that we documented experience-dependent changes in the localization of HDAC4 across the hippocampus and used the whole hippocampus for RNA-seq.

Figure 3i is misleading. They should show the sex differences for all groups, not just the single HDAC5 KO.
We only show sex differences for HDAC5 KO since analysis of all other groups revealed no differences between males and females in the CPP test. Likewise, we found no statistically significant sex differences in all other behavioral assays. We elaborated this point in the figure legend.

Methodological details for counting spines are missing from the manuscript.
We apologize for this oversight and now include the necessary details in the methods.

Clarify what is being refered to when referencing "viral tracing" in the section "HDAC4/5-deficient excitatory circuits have increased synaptic strength".
This clarification is now added, as recommended.

In figure 6, it is not so clear that the nHDAC4 is the HDAC4-3SA. Please indicate this in the figure.
We apologize for the lack of clarity in this figure and alter it, as recommended.

In figure 6, the authors show that HDAC4 stabilization affects memory acquisition but not memory recall. Are there differences in HDAC4 location between the two? It could be nice to show this in the same manner as in Figure 1A.
In these experiments, we used the destabilized genetically encoded HDAC4 as a new tool to repress transcription. The nuclear activity of this fusion protein is acutely controlled by the drug rather than sensory input. The times for TMP delivery were chosen based on quantitative measurements of kinetics of stabilization to ensure that nuclear repression is ON at the time of memory coding or recall.

Gräff & Tsai, 2013, Ann Rev Pharmacol and Toxicol should also be cited when referencing small molecule inhibitors of HDACs in the discussion.
This reference is now included.

Elaborate on how mechanisms of Class I HDACs differ from those of Class IIs.
We included this information in the discussion.

The claim that HDACs4 and 5 have an "irreversible nuclear entry" has been shown to be untrue in Figure 1a.
We believe the reviewer misunderstood this point, perhaps because we did not clearly state it in the text. HDAC4 has been shown to irreversibly accumulate in neuronal nuclei under the pathological conditions, such as Ataxia and Parkinson's disease (see Li et al., 2012; Lang el al., 2018). We clarified this in the text of the revised paper.

The last sentence in the discussion is too strong to comfortably claim and should be toned down as it is not warranted by any data.
We toned down this sentence to refrain from speculating.

Reviewer #2 (Remarks to the Author):
In this manuscript the authors follow up on their 2012 Cell paper to elucidate the functions of the nuclear import of the class II HDACs (4 and 5) in the mouse brain. They generate double excitatory neuronal knockouts of Hdac4/5 as well as a nice tool that lets them rapidly express a triple phospho-mutant form of Hdac4 that stays in the nucleus and acts as a dominant negative. Some of the results in the manuscript are rather expected, such as the nuclear-cytoplasmic translocation of Hdac4 or the evidence Hdac4 acts as a repressor, but it is still nice to see such a complete and well documented dataset showing the role of HDAC4/5 as transient repressors of IEG induction and then using that disruption to suggest the functional importance of IEGs for learning/memory behaviors. Overall the experiments are performed to a high technical standard and the data advance understanding of the mechanisms that sculpt the pattern of activity-inducible gene expression in vivo. I have only very minor concerns.
We thank the reviewer for his/her enthusiastic feedback and helpful suggestions, which we address below and in the revised paper.

The writing could be tempered in places. For example, the references to functions of Hdac4 and Hdac5, which, though perhaps not completely understood, have been published in nice papers from Lisa Monteggia, Chris Cowan, Eric Nestler, and the authors themselves among others, and should not be described as "enigmatic" or "unknown".
We have toned down these statements, as recommended. We used the word "enigmatic" in the original draft since several groups argued that HDAC4 does not function as a nuclear repressor based on lack of changes in gene expression in single

knockout mice. Our results demonstrate that these puzzling observations are attributed to functional redundancy and, also, the fact that repressors only undergo nuclear export in the normal brain in a transient manner.

I was surprised the authors did not discuss HDAC9 since it is the third member of the Class II HDAC family. Is it expressed in the CNS and also undergoes activity-dependent shuttling (e.g. PMID 28729685) and has activity-regulated, MEF2-dependent functions. Is there some reason this factor is not also redundant with Hdac4/5?

We did not examine HDAC9 since it is unlikely that this isoform can compensate for the loss of HDACs 4 and 5, as evidenced by multiple abnormalities of DKO mice which could not be detected in single KOs. Nonetheless, we agree that HDAC9 should be mentioned and do so in the discussion of the revised manuscript.

Given that glutamate neurotransmission and activation of NMDARs leads to nuclear export of HDAC4, it is interesting that the authors find transient increases in nuclear HDAC4/5 following CFC in brain regions showing increased Fos expression, which is a canonical marker of "increased activity". Presumably this reflects differences in the firing of different neurons in the circuit, with those that fire most having cytoplasmic Hdac. However, for readers not familiar with the past literature they may be confused and read this paper to suggest that increased neuronal activity drives the nuclear accumulation of HDAC. This could be made more clear in the text.

We thank the reviewer for noticing this flaw and correct it in the discussion of the revised paper. Indeed, the nuclear translocation of HDAC4 during learning is intriguing, since we and others have previously reported that this protein undergoes nuclear import after NMDAR block. While we are very interested in further elucidating how shuttling of HDAC4 is correlated with network excitation, this question cannot be definitively addressed without simultaneous imaging of HDAC4 and indicators of activity, such as GCaMPs, in intact circuits (also see our response to the next point below). We hope our reviewer will agree that these technically challenging experiments go beyond the scope of this study.

Given that there are changes in synaptic connectivity and function in the double KO mice, the authors should demonstrate that the increased IEG expression they see after CFC is really due to reduced repression of IEGs as opposed to just increased upstream neuronal activity. The easiest way to do this is actually to depolarize the double knockout neurons in culture, since this stimulus bypasses the synapses and directly activates intracellular calcium signaling pathways that normally induce nuclear export of Hdacs. As near as I can tell this experiment is non-redundant with other published experiments because no prior lab has had the double knockout mice to demonstrate the requirement for the Hdacs in the time course or overall magnitude of IEG induction.

We think that, during learning, class IIa HDACs may become transiently de-phosphorylated in neurons that receive strong excitatory input, thereby entering nuclei and restricting transcription in these “engram cells”. If true, the previously documented nuclear import after broad NMDA receptor block would reflect a mechanism by which class IIa HDACs are exported to cytoplasm in steady-state in response to low-frequency and/or spontaneous excitation. Since this model cannot be easily tested in whole animals, as we discussed above, we did not want to speculate in the paper without experimental proof. We also thought to exploit *in vitro* systems for additional analysis of repression. However, wildtype HDACs 4 and 5 are almost exclusively localized in cytoplasm of cultured neurons and, to our surprise, these proteins do not undergo nuclear import after artificial stimulation, most likely due to lack of activation of relevant phosphatases. We attach the representative experiment demonstrating this point (see panel below; we would be happy to include these data in the revised version of the paper, if the reviewer finds it necessary). This limitation of the culture system precludes comparative analyses of repression by native proteins in wildtype and DKO neurons, as opposed to our analysis of constitutively nuclear mutants introduced with lentiviruses. Though we agree that increased synaptic strength in DKO mice may indirectly contribute to observed upregulation of transcription, please note that we do demonstrate that nuclear mutants repress depolarization-induced transcription of an ERG reporter, E-SARE, (Fig. 1) under the conditions that bypass the contribution of activity from synaptic sources.

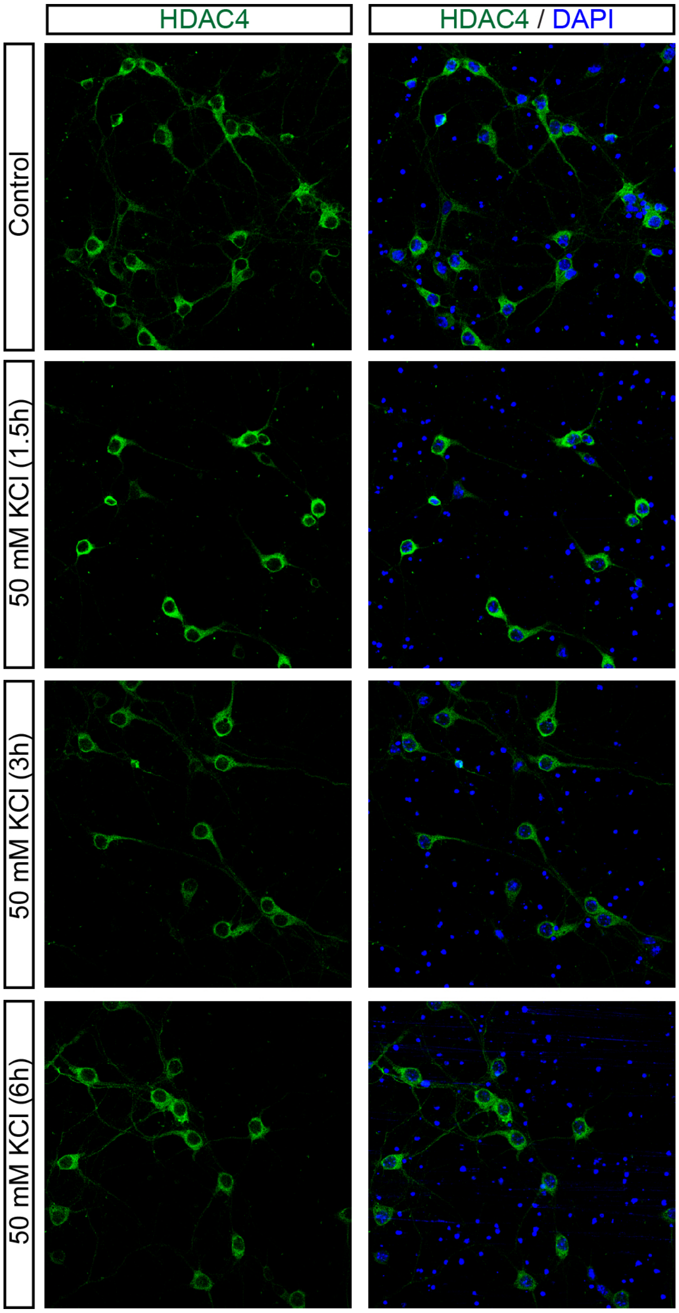

Subcellular localization of wildtype HDAC4 in DIV9 cortical neurons in primary cultures before and after membrane depolarization with KCl.

REVIEWERS' COMMENTS:

Reviewer #1 (Remarks to the Author):

The authors have addressed all our concerns.

Having said so, we still don’t think that they need the single cell dataset in there (and the authors seem to agree that it “is not central to the study”) as we doubt that this dataset is the best way to support their point that “there are no anatomical defects”. Therefore, this dataset is probably best moved to supplementary.

Reviewer #2 (Remarks to the Author):

The authors have addressed all of my concerns.

Zhu et al., Responses to Reviewers' Comments.

Reviewer #1 (Remarks to the Author)

The authors have addressed all our concerns.

Having said so, we still don't think that they need the single cell dataset in there (and the authors seem to agree that it "is not central to the study") as we doubt that this dataset is the best way to support their point that "there are no anatomical defects". Therefore, this dataset is probably best moved to supplementary.

We moved this dataset to the Supplement, as recommended.
